# Supplementary material for: The Salix SmSPR1 Involved in Light-Regulated Cell Expansion by Modulating Microtubule Arrangement
Source: Front Cell Dev Biol. 2019 Nov 28;7:309. doi: 10.3389/fcell.2019.00309 (PMC6892981; doi:10.3389/fcell.2019.00309)
Supplement: Supplementary file 1 [file Data_Sheet_1.pdf]

Supplementary Material

1 Supplementary Figures and

Tables 1.1 Supplementary Figures

```
SmSPR1    MGRGVSAGGGQSSLGYLFG.......SGEA
AtSPR1    MGRGNSCGGGQSSLDYLFGGDAPAPKPVPA

SmSPR1    PKPSTNNAQAAPS.EVQPATNTPPSKPAAA
AtSPR1    PRPAPTESNNGPAPPVTAVTATALTTATTS

SmSPR1    PQPAEINRSVPAGINSTSTNNYMRADGQNA
AtSPR1    VEPAELNKQIPAGIKTP.VNNYARAEGQNT

SmSPR1    GNFITDRPSTKVHAAPGGGSSLGYLFGGGS
AtSPR1    GNFLTDRPSTKVHAAPGGGSSLDYLFTGGK
```

Supplementary Figure 1 | Amino acids sequences comparison between SmSPR1 and AtSPR1. Identical amino acids were marked in green color.

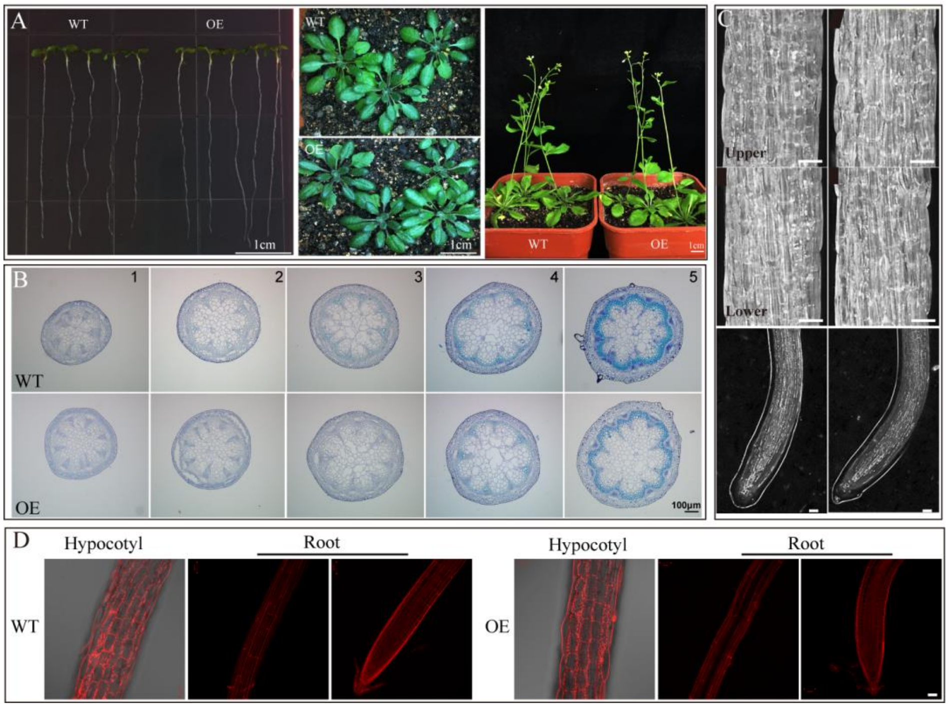

Supplementary Figure 2 | Phenotype of overexpression SmSPR1 in the condition of light. (A) Seedling phenotype between wild-type and *SmSPR1* transgenic plants. **(B)** Stem cross section of wild-type and transgenic Arabidopsis plants. 1-5 show five equal parts from shoot tip of the stem to the base. **(C)** Micrographs of the upper and lower hypocotyl and root. **(D)** PI stain of hypocotyl and root of the wild-type and transgenic seedlings in the presence of light. Bars = 25 μm.

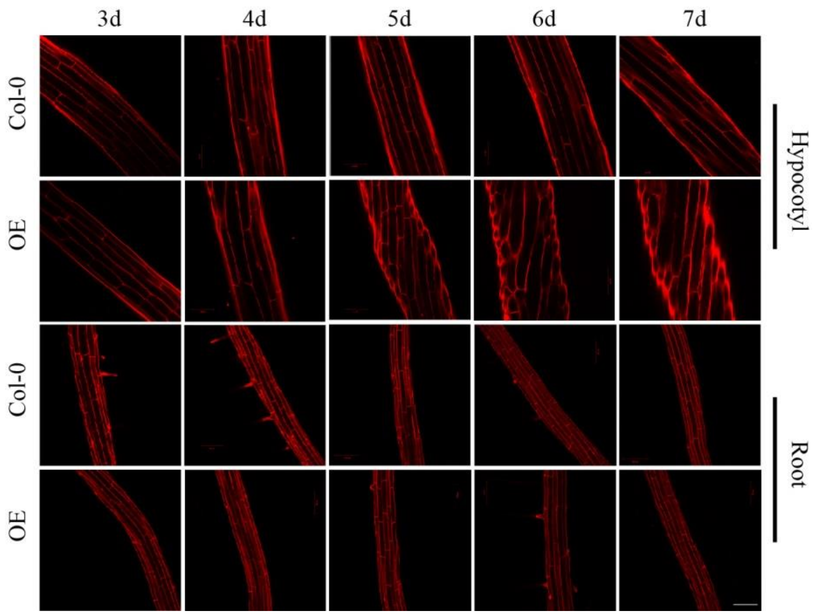

Supplementary Figure 3 | PI staining of hypocotyl and root of the wild-type and transgenic etiolated seedlings from three to seven days of growth. Bars = 100 μm.

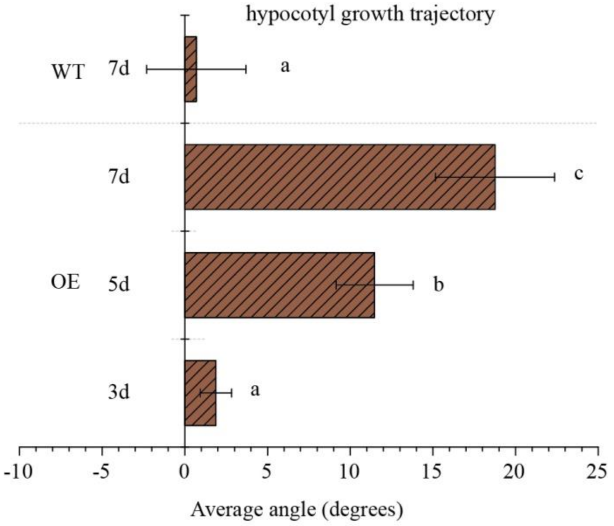

Supplementary Figure 4 | Hypocotyl growth trajectory of wild-type and transgenic etiolated seedlings from three to seven days of growth. Data are expressed as the mean ±SD of > 30 seedlings. Significant differences were determined using the Student’s t test ($P < 0.01$).

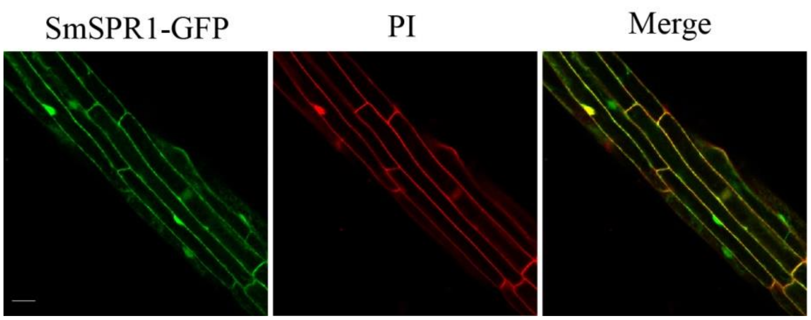

Supplementary Figure 5 | The SmSPR1: GFP localization. (A) Confocal images of SmSPR1: GFP (green). **(B)** PI-stained root (red) in the condition of light. Bars = 20 μm.

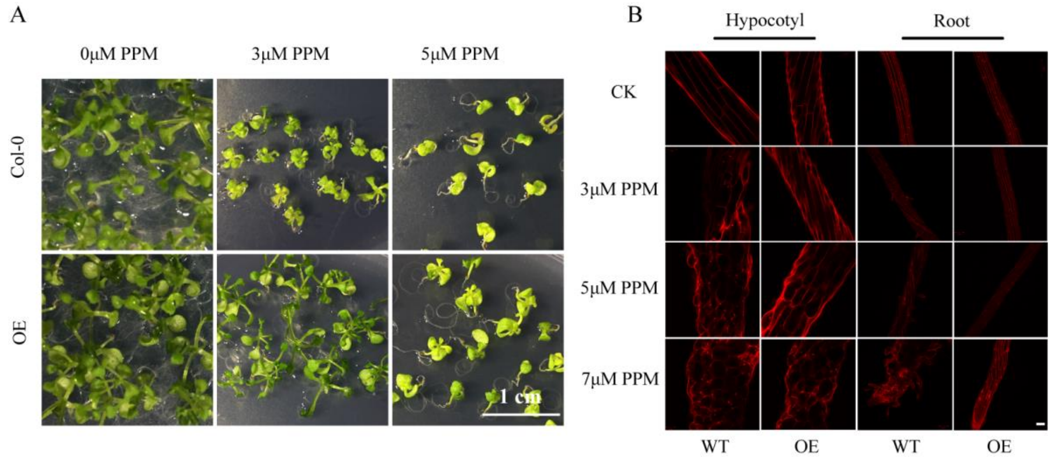

Supplementary Figure 6 | SmSPR1 transgenic seedlings have increased PPM tolerance. (A) Seedling phenotypes of the wild-type and transgenic plants on culture medium containing PPM. **(B)** PI staining of hypocotyl and roots of the wild-type and transgenic plants with the increasing concentrations of PPM in the dark. Bars = 50 μm.

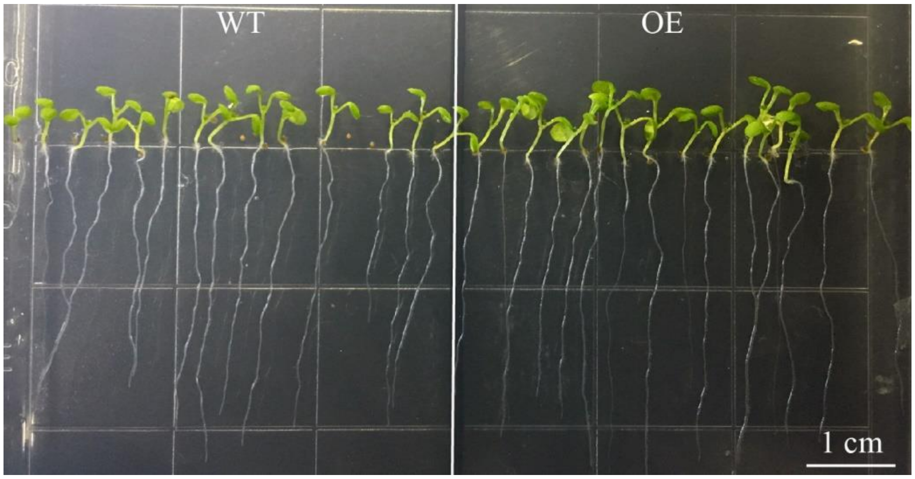

Supplementary Figure 7 | Seedling phenotype in the wild-type and AtSPR1 transgenic plants. Bars = 1 cm.

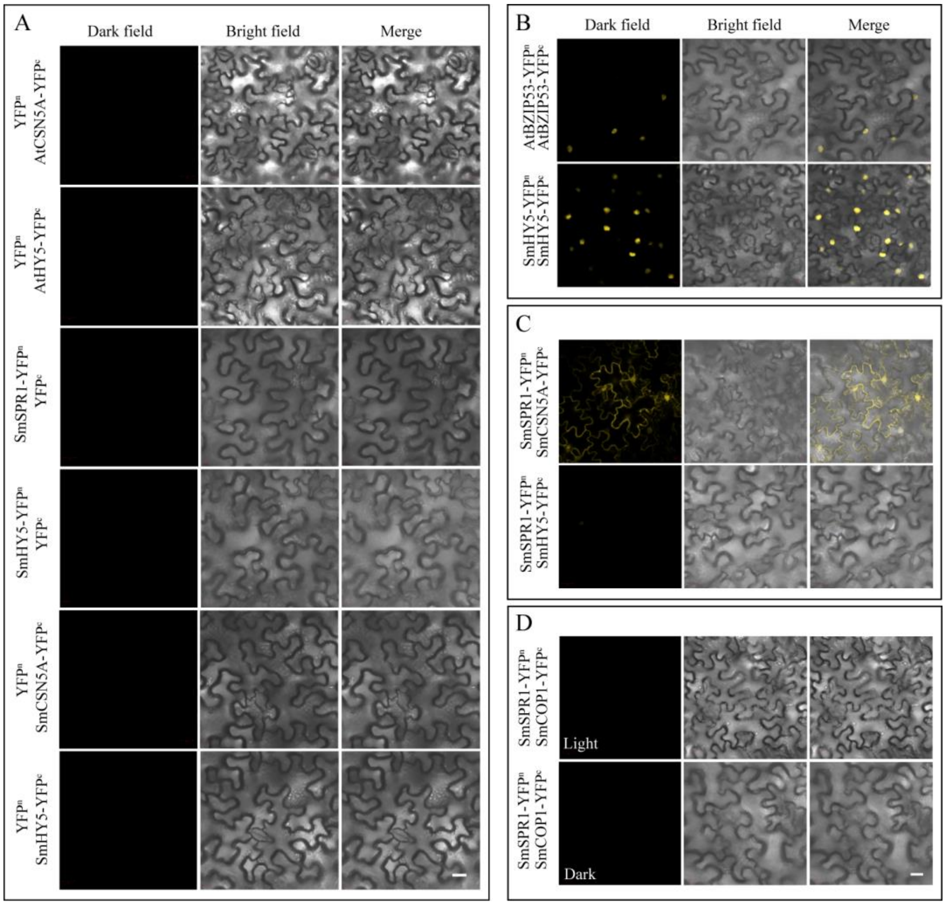

Supplementary Figure 8 | Interactions among SPR1, COP1, and HY5 using the BiFC system. **(A)** Negative control. **(B)** Positive control. **(C)** SmSPR1 interact with CSN5A and HY5 in the dark. **(D)** SmSPR1 interact with COP1 in both light and dark conditions. Bars = 20 μm.

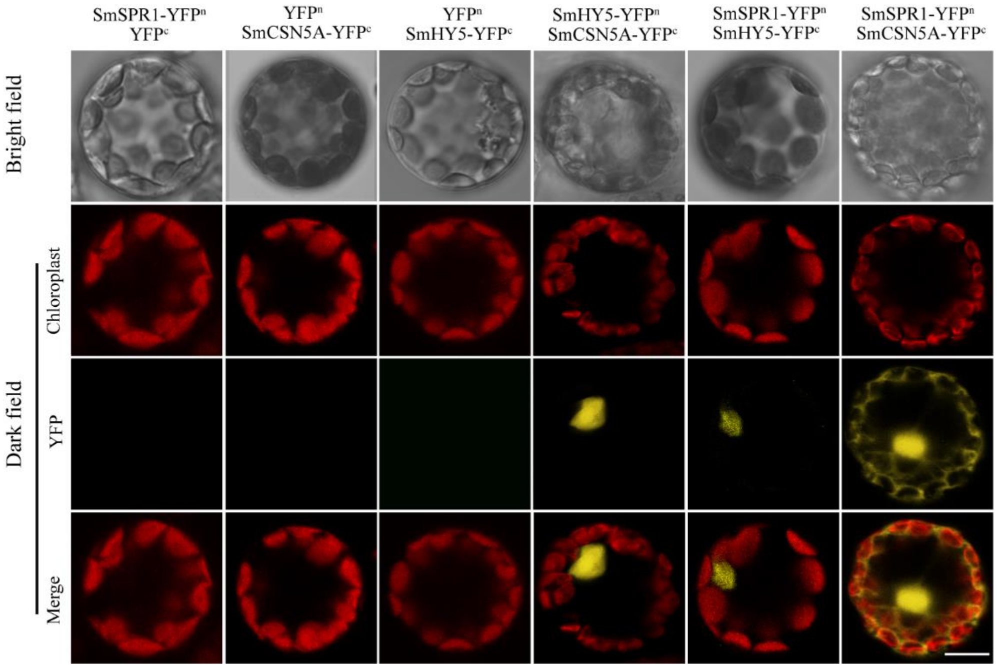

Supplementary Figure 9 | Interactions among SmSPR1, SmCOP1, and SmHY5 in Arabidopsis protoplasts using BiFC assay. Bars = 10 μm.

1.2 Supplementary tables

Supplementary Table 1 | List of primers used for cloning *SmSPR1*, *SmCSN5A*, *SmHY5* and *SmCOP1*

Gene	Forward (F) and reverse (R) primers (5' to 3')
SmSPR1	F: ATGGGTCGTGGAGTTAGCGC<br>R: TCAGTTGCTACCACCACCAAAGA
SmSPR1L1	F: ATGAGTAGAGGTGGGAGCTAC<br>R: TCACTTATCTCCAAACAGGT
SmSPR1L2	F: ATGAGTAGAGGTGGGAGCTT<br>R: TCACTTATCTCCAAATAGGT
SmSPR1L3	F: ATGGGTCGAGGAGTTAGCGCTGG<br>R: TCAGGCATGCTGATTTGTCAAAG
SmSPR1L4	F: ATGGGGCGTGGAGTGAGCAG<br>R: TCATTTCCCACCACCAGCAC
SmSPR1L5	F: ATGGGTCGTGGAGTCAGTGC<br>R: GTGCTGGTGGTGGGAAATGA
SmCSN5A	F: ATGGATCCCTACCCTTCTTCTTC<br>R: TCATGTTTCAACCATTGGCTCAGG
SmHY5	F: ATGCAAGAACAAGCAACCAGTTC<br>R: TCACGAAGAACCATCTGCATTAGCAT
SmCOP1	F: ATGGAAGAGGTTTCAACGGG<br>R: AGCTGCAAGAACCAATACTTTTATT

Supplementary Table 2 | List of primers used for Semi-quantitative RT-PCR of *SmSPR1* and *AtSPR1*

Gene	Forward (F) and reverse (R) primers (5' to 3')
SmSPR1	F: CTGTTTGGGAGTGGAGAGGCT<br>R: CACCAAAGAGGTATCCCAGAGAA
AtSPR1	F: TCCCGCTCCTACTGAGTCTAAC<br>R: AGTTGCCGGTGTTCTGTCCTTC
AtSPR1L1	F: TAACAATGCTCCTGCACCATCA<br>R: GTCCGTAAGGAAGTTGCCTGTG
AtSPR1L2	F: ACTCAAGCTGCTGCTGCAAACG<br>R: CCACCTCCTGGAGCTGAGTGAA
AtSPR1L3	F: GATAACCACCACGACGACCACA<br>R: ACCAGGAGCTGCATGAACCTTA
AtSPR1L4	F: ATGGAGCTGGAGGTAGACCCAAGA<br>R: GTAGATGGTCGTTCCGTGAGGA
AtSPR1L5	F: TAGCCCCAAAGCCTGCACCA<br>R: AGGAACCGATTTCACCTTCGTC
At18S	F: CGGCTACCACATCCAAGGAA<br>R: GCTGGAATTACCGCGCGGCT

Supplementary Table 3 | List of primers used for real-time RT-PCR analysis

Gene	Forward (F) and reverse (R) primers (5’ to 3’)
qSmSPR1	F: GAGTTCATTGGGCTATCTGTTTGG<br>R: GCAGCAGGTTTAGAAGGAGGTG
qSmSPR1L1	F: TGACATTACCACGGAGAACCC<br>R: TCCTGGCACTGACTTGACTT
qSmSPR1L2	F: CAGAGTTCTTTGGGCTACCT<br>R: ATAGTTGTTTGAGACCTGCTTC
qSmSPR1L3	F: AGCCTGCAAACAATCCACCTC<br>R: TGCCAGCGTTCTGACCATCT
qSmSPR1L4	F: CCTGTAACCAATGAGCCTGTAGC<br>R: GCCCATCAGCACGGAAATAG
qSmSPR1L5	F: TGCGAGCATTAGCCCTTCT<br>R: TTGCCACAGTTCTGTCCATCT
qSmGAPDH	F: GGATAAGGCTGCTGCTCACT<br>R: TCTGGTGTGTAACTCTTCTC

Supplementary Table 4 | List of primers used for overexpression conduct SmSPR1 and AtSPR1

Gene	Forward (F) and reverse (R) primers (5' to 3')
OE-SmSPR1	F:CGGGATCCATGGGTCGTGGAGTTAGCGC<br>R:ACGCGTCGACTCAGTTGCTACCACCACCAA
OE-SmSPR1-attB	F:GGGGACAAGTTTGTACAAAAAAGCAGGCTTCATGGGTCGTGGAGTTAGCGC<br>R:GGGGACCACTTTGTACAAGAAAGCTGGGTCTCAGTTGCTACCACCACCAAAG
OE-AtSPR1-attB	F:GGGGACAAGTTTGTACAAAAAAGCAGGCTTCATGGGTCGTGGAAACAGCTGT<br>R:GGGGACCACTTTGTACAAGAAAGCTGGGTCTTACTTGCCACCAGTGAAGAG

Supplementary Table 5 | List of primers used for construction of prokaryotic expression vector

**Gene**	**Forward (F) and reverse (R) primers (5' to 3')**
28a-SmSPR1	F: GTCGACTCATGGGTCGTGGAGTTAG<br>R: GCGGCCGCGTTGCTACCACCACC
21a-HY5	F: CGCCATATGCAAGAACAAGCAACCAGTTC<br>R:ATGATCTTTATAATCACCGTCATGGTCTTTGTAGTCCGAAGAACCATCTGCATTAGCA
21a-CSN5A	F: CGGAATTCATGGATCCCTACCCTTCTTCTTC<br>R1:ATGATCTTTATAATCACCGTCATGGTCTTTGTAGTCTGTTTCAACCATTGGCTCAG<br>R2:CCGCTCGAGTCACTTGTCATCGTCATCCTTGTAGTCGATGTCATGATCTTTATAATCACCGTCA

Supplementary Table 6 | List of primers used for Yeast Two-Hybrid

Gene	Forward (F) and reverse (R) primers (5' to 3')
Y-SmSPR1	F: AAGGCCATTACGGCCATGGGTCGTGGAGTTAGCGC<br>R: CCGGCCGAGGCGGCCTCATCAGTTGCTACCACCACCA
Y-CSN5A	F: AAGGCCATTACGGCCATGGATCCCTACCCTTCTTCT<br>R: CCGGCCGAGGCGGCCTCATGTTTCAACCATTGGCTC
Y-HY5	F: AAGGCCATTACGGCC ATGCAAGAACAAGCAACCAG<br>R: CCGGCCGAGGCGGCCTCA CGAAGAACCATCTGCAT
Y-COP1	F: AAGGCCATTACGGCCATGGAAGAGGTTTCAACGGG<br>R: CCGGCCGAGGCGGCCTCA AGCTGCAAGAACCAATA

Supplementary Table 7 | List of primers used for BiFC.

Gene	Forward (F) and reverse (R) primers (5' to 3')
BiFC-SmSPR1	F:GGGGACAAGTTTGTACAAAAAAGCAGGCTTCATGGGTCGTGGAGTTAGCGC<br>R:GGGGACCACTTTGTACAAGAAAGCTGGGTCGTTGCTACCACCACCAAAGAG
BiFC-SmCSN5A	F:GGGGACAAGTTTGTACAAAAAAGCAGGCTTCATGGATCCCTACCCTTCTTCTTC<br>R:GGGGACCACTTTGTACAAGAAAGCTGGGTCTGTTTCAACCATTGGCTCAGG
BiFC-SmCOP1	F:GGGGACAAGTTTGTACAAAAAAGCAGGCTTCATGGAAGAGGTTTCAACGGG<br>R:GGGGACCACTTTGTACAAGAAAGCTGGGTCAGCTGCAAGAACCAATACTTTTATT
BiFC-AtCSN5A	F:GGGGACAAGTTTGTACAAAAAAGCAGGCTTCATGGAAGGTTCCTCGTCAGC<br>R: GGGGACCACTTTGTACAAGAAAGCTGGGTC CGATGTAATCATGGGCTCTGG
BiFC-SmHY5	F:GGGGACAAGTTTGTACAAAAAAGCAGGCTTCATGCAAGAACAAGCAACCAGTTC<br>R:GGGGACCACTTTGTACAAGAAAGCTGGGTCCGAAGAACCATCTGCATTAGCAT
BiFC-AtHY5	F:GGGGACAAGTTTGTACAAAAAAGCAGGCTTCATGCAGGAACAAGCGACTAGC<br>R:GGGGACCACTTTGTACAAGAAAGCTGGGTCAAGGCTTGCATCAGCATTAGA
BiFC-AtBZIP53	F:GGGGACAAGTTTGTACAAAAAAGCAGGCTTCATGGGGTCGTTGCAAATGCA<br>R:GGGGACCACTTTGTACAAGAAAGCTGGGTCGCAATCAAACATATCAGCAGAAGC
